# Optimization of Cervical Cancer Screening: A Stacking-Integrated Machine Learning Algorithm Based on Demographic, Behavioral, and Clinical Factors

**DOI:** 10.3389/fonc.2022.821453

**Published:** 2022-02-15

**Authors:** Lin Sun, Lingping Yang, Xiyao Liu, Lan Tang, Qi Zeng, Yuwen Gao, Qian Chen, Zhaohai Liu, Bin Peng

**Affiliations:** ^1^ School of Public Health and Management, Chongqing Medical University, Chongqing, China; ^2^ Department of Obstetrics, The First Affiliated Hospital of Chongqing Medical University, Chongqing, China; ^3^ Department of Physical Examation, The First Affiliated Hospital of Chongqing Medical University, Chongqing, China; ^4^ Information Section, The First Affiliated Hospital of Chongqing Medical University, Chongqing, China

**Keywords:** machine learning, cervical cancer, risk, artificial intelligence, personalized screening

## Abstract

**Purpose:**

The purpose is to accurately identify women at high risk of developing cervical cancer so as to optimize cervical screening strategies and make better use of medical resources. However, the predictive models currently in use require clinical physiological and biochemical indicators, resulting in a smaller scope of application. Stacking-integrated machine learning (SIML) is an advanced machine learning technique that combined multiple learning algorithms to improve predictive performance. This study aimed to develop a stacking-integrated model that can be used to identify women at high risk of developing cervical cancer based on their demographic, behavioral, and historical clinical factors.

**Methods:**

The data of 858 women screened for cervical cancer at a Venezuelan Hospital were used to develop the SIML algorithm. The screening data were randomly split into training data (80%) that were used to develop the algorithm and testing data (20%) that were used to validate the accuracy of the algorithms. The random forest (RF) model and univariate logistic regression were used to identify predictive features for developing cervical cancer. Twelve well-known ML algorithms were selected, and their performances in predicting cervical cancer were compared. A correlation coefficient matrix was used to cluster the models based on their performance. The SIML was then developed using the best-performing techniques. The sensitivity, specificity, and area under the curve (AUC) of all models were calculated.

**Results:**

The RF model identified 18 features predictive of developing cervical cancer. The use of hormonal contraceptives was considered as the most important risk factor, followed by the number of pregnancies, years of smoking, and the number of sexual partners. The SIML algorithm had the best overall performance when compared with other methods and reached an AUC, sensitivity, and specificity of 0.877, 81.8%, and 81.9%, respectively.

**Conclusion:**

This study shows that SIML can be used to accurately identify women at high risk of developing cervical cancer. This model could be used to personalize the screening program by optimizing the screening interval and care plan in high- and low-risk patients based on their demographics, behavioral patterns, and clinical data.

## Introduction

Cervical cancer is one of the most common malignant tumors in women worldwide (1). The 5-year survival rate for early-stage cervical cancer is high, ranging from 80% to 90% (2). However, the cure rate goes down to 10% for stage 4 disease (3). Cervical screening has, therefore, an important role in identifying the disease at an early stage and hence reduces the morbidity and mortality from the disease. The incidence and mortality from cervical cancer vary across different countries and tend to be lower in highly developed countries due to well-established screening and vaccination programs (4). However, underdeveloped regions often do not have sufficient medical resources allocated to screening. This implies that there is an increased need to identify women at a high risk of developing cervical cancer to optimize the screening interval and hence make better use of medical resources (5, 6).

Parametric prediction models can be used to better identify the early risk warning signs of cervical cancer (7–9). However, to our knowledge, there is currently no comprehensive risk prediction model based on demographic information, behavioral habits, and medical history for cervical cancer. Prediction models need to be able to make use of individual information to accurately predict the risk of developing the disease. Artificial intelligence (AI) and machine learning (ML) can be used to analyze large volumes of data to make accurate predictions and to identify hidden interactions (10, 11). Therefore, the use of AI and ML in the medical field has increased exponentially during the past few years. However, current risk prediction models for cervical cancer are based on former-generation algorithms, such as the decision tree model and random forest (RF) (12). Until recently, more powerful algorithms such as the stacking-integration machine learning (SIML) have yet to be fully explored. SIML’s automatic large-scale integration strategy can effectively combat overfitting by adding regular items and transferring the integrated knowledge to a simple classifier, which is the best way to improve the effectiveness of machine learning.

This study aimed to develop an SIML that could be used to identify women at a high risk of developing cervical cancer based on their demographic, behavioral, and medical history and hence personalize the screening program according to their risk factors.

## Materials and Methods

### Study Populations

**Figure 1:**
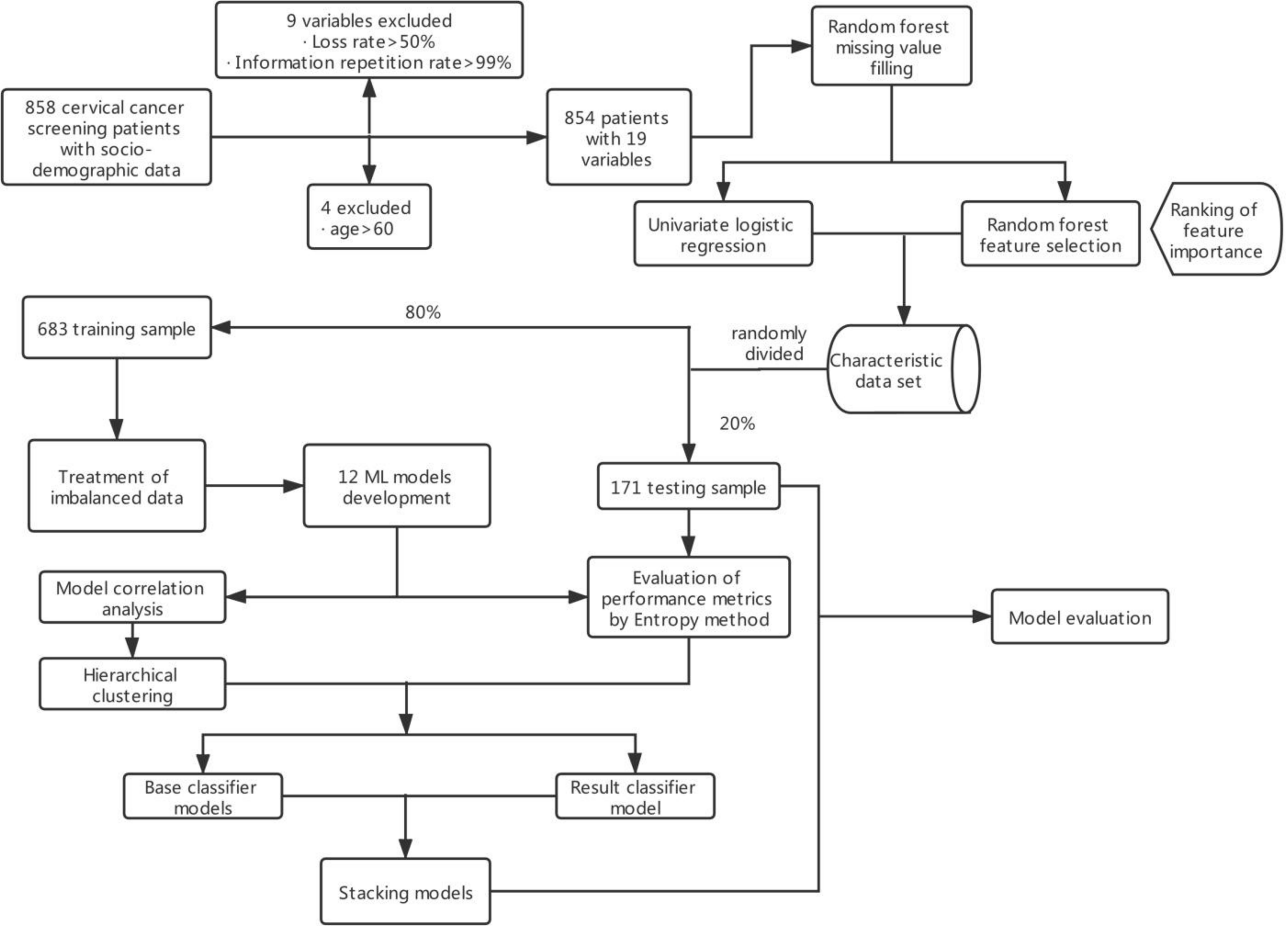
Flowchart illustrating the development and validation of ML models. ML, machine learning.

These data were obtained from the public dataset provided by Kelvin Fernandes in the UCI database. The data were based on early screening data for cervical cancer collected at the *Hospital Universitario de Caracas*, Venezuela, from March 2012 to September 2013 (13). The majority of patients were of low socioeconomic status, low income, and low educational level. The patients were aged 13–84 years, with an average age of 27 years, and 88.6% of them had at least one pregnancy. The data collected included demographics, behavioral patterns, and medical histories of 858 patients. A total of 18 different potential risk variables were identified and coded, as shown in **Supplementary Table S1**. Due to missing variables for privacy concerns, not all patient variables were available for analysis. Feature datasets excluded variables with more than half loss rate or those that have all identical values. The original general data parameter index code is available in **Supplementary Table S1**, and the main content of the modeling is shown in **Figure 1**.

### Dataset Preprocessing

The premise of an efficient and reliable disease risk prediction model was the accuracy of the data. Visualization of the data was first performed using the public packages related to ML in R, version 3.6.0 (The R Foundation for Statistical Computing, Vienna, Austria), while the PRISM software version 7.0 for Windows (GraphPad Software Inc., San Diego, CA, USA) was used to plot the data (**Supplementary Figure S1**).

Following visualization of the data, 18 high-risk prediction features (**Supplementary Table S1**) of a positive biopsy were identified. Continuous variables were categorized as follows. The ages of the patients were grouped into four categories: below 20 years, 20–29 years, 30–44 years, and 45–60 years, while the age of first sexual intercourse was grouped into five groups: below 13 years, 13–15 years, 16–17 years, 18–19 years, and above 20 years. Other classification variables were input according to the original characteristics.

Not all the data for each predictive feature were available. About 20%–30% of the clinical predictive data and about 0%–15% of the behavioral data were missing. The missing part of the data had to be estimated by using the information available in the existing data to replace the missing data with values (14). However, due to a large number of missing data, conventional mean and median filling methods could not be used in this case, since these techniques cannot guarantee data authenticity because the filling values are mostly unreal values, which will affect the accuracy of model construction. Therefore, nonparametric missing value imputation using RF (MissForest) was used to process missing data as suggested by Stekhoven et al. (15). The parameters of the model were set as follows: the maximum iterations were set to 10. The number of trees was chosen to be 100.

### Feature Selection

The model was designed to rely on a limited and effective set of features that do not require excessive input from patients. Using the RF model, a total of 18 predictors for developing cervical cancer were identified. The univariate logistic regression and feature selection model were then used to quantify the odds ratio (OR) and the contributing risk of each predictive value for developing cervical cancer. The analysis of feature selection was based on the RF classifier, whereby the importance of each predictive feature was sorted by using the error rate measurement. Specifically, for each tree in the RF, the error rate for classification of the out-of-bag portion of the data was recorded. The feature importance score was calculated by estimating improvement in the classification error rate of each feature. Finally, the importance scores of all trees in RF were averaged to get the final score of each feature (16). Nine important predictive features were finally identified.

### Treatment of Imbalanced Data

Imbalanced data refer to the uneven distribution of data among different categories, whereby the main categories have a much larger representation (17). The imbalance ratio (IR) is expressed as the ratio of the number of large sample categories to the number of small sample categories. A large IR generally has a negative impact on the classification effect of the model and can lead to an inaccurate classification.

Two techniques were used to deal with imbalanced data in our study. The first method involved the use of resampling based on samples (oversampling, undersampling, and hybrids). The other method combines the use of resampling methods *via* the random oversampling example (ROSE) (18) and synthetic minority oversampling technique (SMOTE) (19) algorithms. In this study, five different resampling methods and RF were combined to build the models, and ultimately the best method was selected and integrated into the final SIML.

### Model Development

Following class imbalance treatment, the cervical cancer screening data were randomly assigned to the training dataset (80% of data) and testing dataset (20% of the data). The training dataset was used to develop the algorithm, while the testing dataset was used to evaluate the performance of the algorithm. We then selected 12 widely used ML algorithms including RF, Stochastic Gradient Boosting (SGB), Bagged Classification and Regression Tree (TreeBag), eXtreme Gradient Boosting (XGBoost), Monotone Multi-Layer Perceptron Neural Network (MonMLP), Support Vector Machines with Radial Basis Function Kernel (SVMRadial), K-Nearest Neighbors (KNN), Gaussian Process with Radial Basis Function Kernel (GaussPrRadial), Regularized Logistic Regression (RgeLogistic), Stabilized Linear Discriminant (SLDA), AdaBoost Classification Trees (AdaBoost), and Logistic Model Trees (LMT). All of these supervised algorithms were implemented using the free and open-source library caret in R3.6.0. To adjust the optimal tuning parameters of each ML algorithm, we used 10-fold cross-validation and repeated three times on the training set. This method involved dividing the training set into 10 sets and using nine sets for training and the remaining set was used for verification. This was performed 10 times, and the results of the different test sets were averaged, ensuring an independent result from the actual dataset subdivision (20).

RF, TreeBag, SGB, AdaBoost, and XGBoost are integrated algorithms that combine multiple simple tree models (21, 22) and are considered to be the most accurate for making predictions using various datasets for several applications. MonMLP is a feed-forward Artificial Neural Network (ANN) model, which maps multiple input datasets to a single output. As a popular ML algorithm, MonMLP has incomparable advantages in prediction accuracy. However, it requires tuning of many parameters and a large number of data for training (23). SVMRadial is an SVM model with Radial Basis Function, which constructs a decision curve in high-dimensional feature space to perform binary classification (24). KNN, GaussPrRadial, RegLogistic, and SLDA are relatively efficient and effective simple classification algorithms in data mining. Although these algorithms are relatively simple, they still perform very well and result in a model that is easier to interpret (21, 25). LMT is an algorithm generated by the combination of linear logistic regression and decision tree induction. It has been proven to be an accurate and simple classifier, which is also competitive with other advanced classifiers (such as RF) and easier to explain (26).

**Figure 2:**
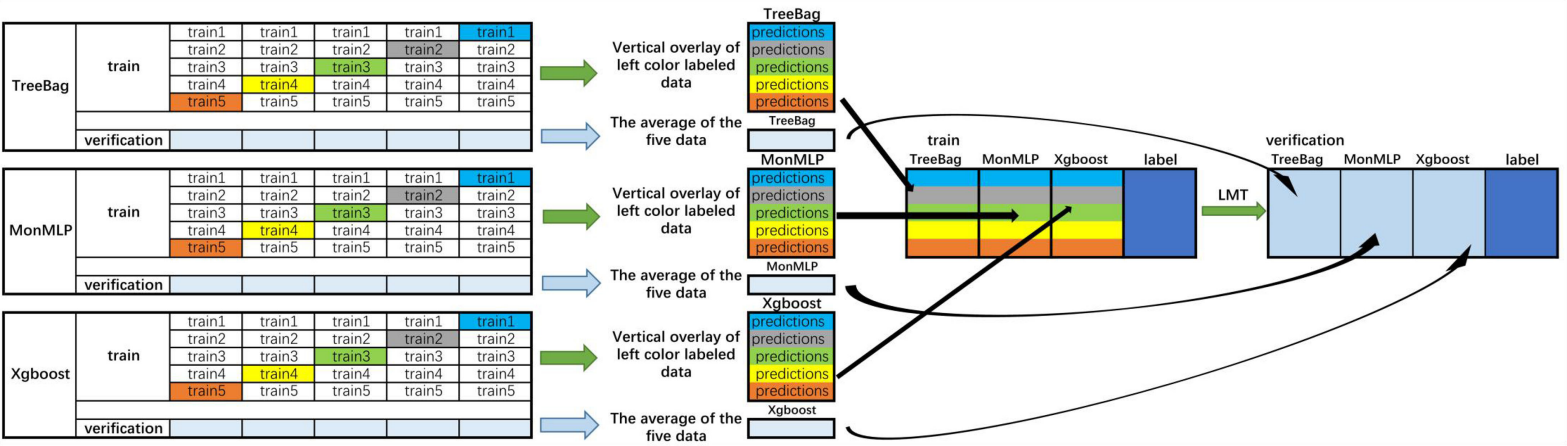
Flowchart of the integrated stacking structure. 1) The training sets were divided into two groups of data: training and verification sets, and the training set is divided into five equal parts. 2) Take TreeBag as an example (The Figures above are Treebag, MonMLP, and XGBoost); train1, train2, train3, train4, and train5 are used as verification sets in proper sequence, and the rest are used as training sets. The model is trained by 5-fold cross-validation, and then predicted on the test set. Therefore, TreeBag can get five prediction results, which are vertically overlapped and merged into a matrix. The other two models are the same. 3) The predicted values of the three models are taken as three characteristic variables, and the resulting classifier LMT is used for fitting. Then, the reserved training set was averaged. The verification set of each characteristic variable was used to verify the performance of the LMT-stacking model. TreeBag, Bagged Classification and Regression Tree; MonMLP, Monotone Multi-Layer Perceptron Neural Network Random Over-Sampling Examples; XGBoost, eXtreme Gradient Boosting; LMT, Logistic Model Trees.

The performances of the algorithms were compared to select the optimal stacking algorithm. Stacking is a common integrated learning framework in the Kaggle competition, integrating many models to improve the result prediction accuracy. It is generally used to train a two-layer learning structure. The first layer (known as the learning layer) trains *n* different classifiers, and their predicted results are combined into a new feature set, which is then used as the input of the next layer classifier (27) (**Figure 2**). Stacking has the characteristics of distributing multiple classifiers while ensuring excellent performance. In summary, the stacking-integrated learning framework has two requirements for base classifiers: large differences between classifiers and high accuracy of classifiers. However, it is prone to overfitting (28). The features of the second layer come from learning the results of the first one. Thus, the original features should not be included in the features of the data of the second layer to reduce the risk of overfitting. The best choice of the second layer classifier is a relatively simple classifier. RegLogistic is a better method in Stacking (29), but LMT is more robust in overfitting (26), and can therefore be used instead.

### Model Comparisons

The optimal tuning parameters of each ML algorithm were determined by cross-validation on the training samples after imbalance data processing. The models’ internal verification scores were obtained from the training dataset, while the external validation scores were obtained from the test sets. External validation scores could be used to test the generalization power of the model. The performance evaluation of binary data (positive vs. negative) was mainly based on the sensitivity $\left(\frac{TP}{TP+FN}\right)$ and specificity $\left(\frac{TN}{TN+FP}\right)$ , where TP, FP, TN, and FN represent the number of true positives, false positives, true negatives, and false negatives, respectively. The area under the curve (AUC) was used to reflect the relationship between two performance variables. F1 scores and F2 scores were also used to measure the model’s accuracy.

$F1=\frac{2*precision*recall}{precision+recall},\;F2=\frac{(1+2^{2})*precision*recall}{2^{2}*precision+recall},\;in\;which\;precision=\frac{TP}{TP+FP}\;and\;recall=\frac{TP}{TP+FN}$

Alternatively, the F1 score and F2 score were a kind of harmonic mean of model accuracy and recall (30), comparing different model performances in identifying true disease predictions when compared to false positives. The weight of the F2 score was more inclined to the recall value of the model and focuses on the sensitivity index of the model.

The entropy weight method was an objective weighting method that can be used to reduce the influence of human factors. After averaging the seven performance metrics of the 12 models, we calculated the weights of each metric using the entropy weight method (**Supplementary Table S2**).

The base models in the stacking structure were selected to be independent and weakly correlated. The correlation coefficients between the 12 models were calculated, and the correlation coefficient matrix was used to cluster the model by hierarchical clustering. Each cluster selected a classifier with the best performance as the base model.

## Results

### Study Participants

The baseline characteristics of the participants are summarized in **Table 1**. Among the 858 screened patients, 4 (0.46%) were excluded, as they were over 60 years old. The majority of the included cases (46.14%) were aged between 20 and 29 years, 31.38% had their first sexual intercourse between 13 and 15 years old, 15.69% of the patients were smokers, 68.97% of patients took hormonal contraceptives, and 9.25% of the patients had sexually transmitted disease. However, only 6.44% of the performed biopsies were positive.

**Table 1:** Sociodemographic factors associated with cervical cancer: univariate logistic regression analysis.

	Total (n = 854)	Biopsy negative (n = 799)	Biopsy positive (n = 55)	p	Odds ratio (95% CI)
**Age, years**				**0.045**	
<20	179 (20.96)	173 (21.65)	6 (10.91)	Referent	
**20–29**	**394 (46.14)**	**366 (45.81)**	**28 (50.91)**	**0.085**	**2.206 (0.897–5.426)**
30–44	262 (30.68)	245 (30.66)	17 (30.91)	0.153	2.001 (0.773–5.178)
**45–60**	**19 (2.22)**	**15 (1.88)**	**4 (7.27)**	**0.004**	**7.689 (1.952–30.281)**
Number of sexual partners	2.00 (2.00–3.00)	2.00 (2.00–3.00)	2.00 (2.00–3.00)	0.986	1.001 (0.850–1.180)
**First sexual intercourse(age), years**				**0.061**	
<13	11 (1.29)	9 (1.13)	2 (3.64)	Referent	
**13–15**	**268 (31.38)**	**256 (32.04)**	**12 (21.82)**	**0.063**	**0.211 (0.041–1.085)**
16–17	271 (31.73)	252 (31.54)	19 (34.55)	0.186	0.339 (0.068–1.683)
18–19	199 (23.30)	180 (22.53)	19 (34.55)	0.363	0.475 (0.096–2.361)
**≥20**	**105 (12.30)**	**102 (12.77)**	**3 (5.45)**	**0.038**	**0.132 (0.020–0.898)**
**Num of pregnancies**	**2.00 (1.00–3.00)**	**2.00 (1.00–3.00)**	**3.00 (1.00–4.00)**	**0.071**	**1.180 (0.986–1.413)**
Smoking, yes	134 (15.69)	123 (15.39)	11 (20.00)	0.365	1.374 (0.690–2.734)
	**(n = 134)**	**(n = 123)**	**(n = 11)**		
Smoking (years)	7.00 (2.00–11.00)	6.67 (2.00–11.00)	10.00 (3.00–15.00)	0.100	1.062 (0.988–1.141)
Smoking (packs/year)	1.38 (0.51–3.00)	1.35 (0.51–3.00)	2.00 (1.25–3.40)	0.169	1.017 (0.910–1.137)
Hormonal contraceptives, yes	589 (68.97)	553 (69.21)	36 (65.45)	0.561	0.843 (0.474–1.499)
	**(n = 589)**	**(n = 553)**	**(n = 36)**		
**Hormonal Contraceptives(years)**	**2.00 (1.00–5.00)**	**2.00 (1.00–4.50)**	**1.50 (0.50–9.50)**	**0.007**	**1.092 (1.024–1.165)**
IUD, yes	199 (23.30)	187 (23.40)	12 (21.82)	0.788	0.913 (0.472–1.768)
	**(n = 199)**	**(n = 187)**	**(n = 12)**		
IUD (years)	2.19 (1.60–3.77)	2.17 (1.56–3.65)	3.00 (2.50–4.88)	0.352	1.081 (0.918–1.272)
STDs, yes	79 (9.25)	67 (8.39)	12 (21.82)	0.395	2.000 (0.406–9.886)
	**(n = 79)**	**(n = 67)**	**(n = 12)**		
Number of STDs	2.00 (1.00–2.00)	2.00 (1.00–2.00)	2.00 (1.00–2.00)	0.926	0.958 (0.388–2.365)
STDs: condylomatosis	44 (55.70)	37 (55.22)	7 (58.33)	0.842	1.135 (0.327–3.941)
STDs: vaginal condylomatosis	4 (5.06)	4 (5.97)	0 (0.00)	/	/
STDs: vulvo-perineal condylomatosis	43 (54.43)	36 (53.73)	7 (58.33)	0.768	1.206 (0.347–4.183)
STDs: syphilis	18 (22.78)	18 (26.87)	0 (0.00)	/	/
STDs: HIV	18 (22.78)	13 (19.40)	5 (41.67)	0.100	2.967 (0.811–10.861)

Portions in bold represent p < 0.1. IUD, intrauterine device; STD, sexually transmitted disease.

### Predictors for a Positive Biopsy

The result of the univariate logistic regression analysis evaluating the relationship between behavioral habits, medical history, and positive biopsy is summarized in **Table 1**. The p values of age (p = 0.045), first sexual intercourse (age) (p = 0.061), number of pregnancies (p = 0.071), and use of hormonal contraceptives (years) (p = 0.007) were less than 0.1, suggesting a relationship to the occurrence of cervical cancer. Among them, the risk of cervical cancer was significantly higher in the 45–60 age group when compared with those under 20 years old (OR = 7.689, 95% CI: 1.952–30.281). Compared with those less than 13 years old for the first intercourse, the risk of cervical cancer was significantly lower in people who had sex for the first time after the age of 20 (OR = 0.132, 95% CI: 0.020–0.898). The longer use of hormonal contraceptives and a larger number of pregnancies were also features associated with an increased risk of developing cervical cancer, with ORs of 1.092 (95% CI: 1.024–1.165) and 1.180 (95% CI: 0.986–1.413) respectively.

**Figure 3:**
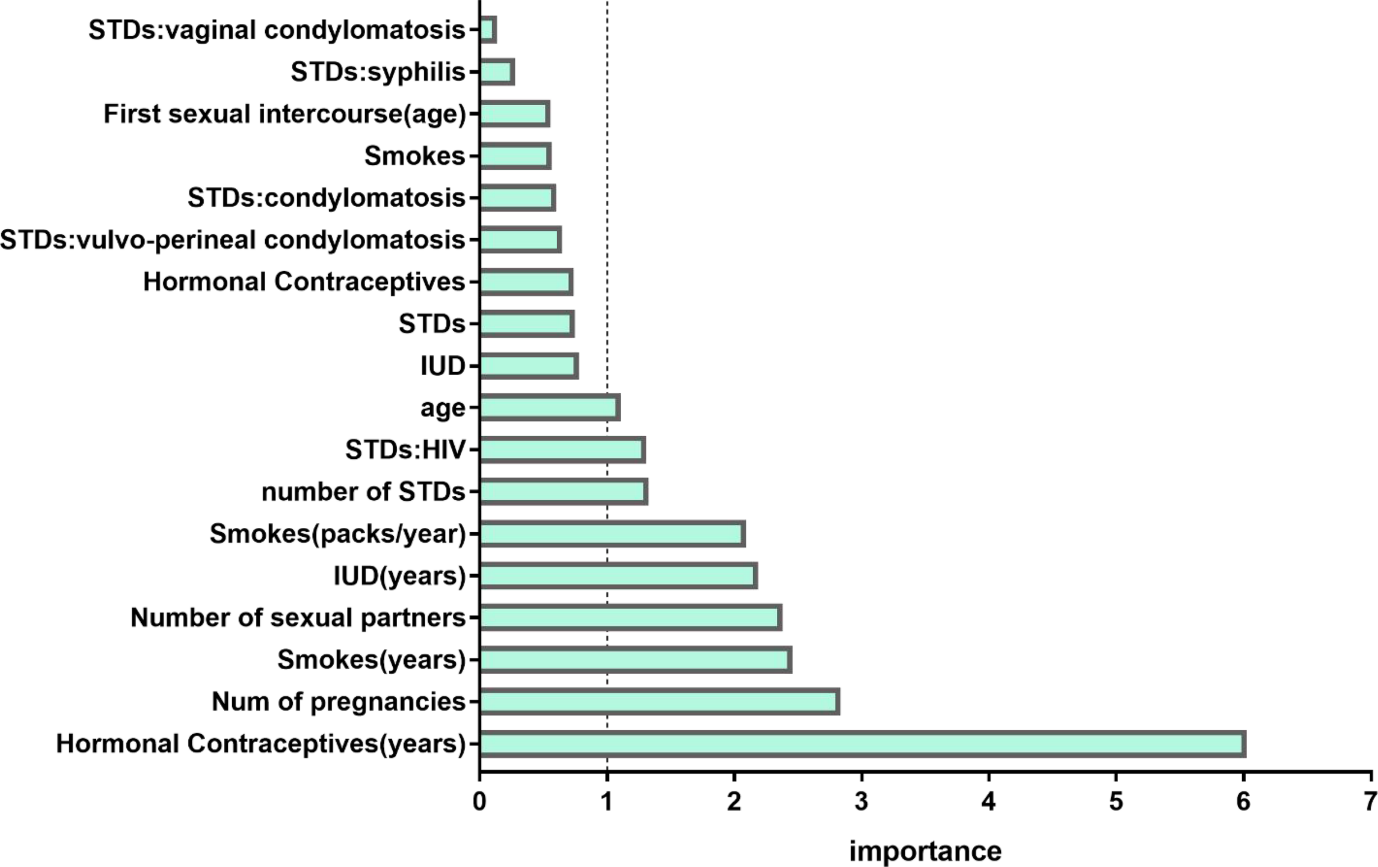
Variable importance measures for each predictor of morbidity. IUD, intrauterine device; STD, sexually transmitted disease; HIV, human immunodeficiency virus.

The feature selection method using RF was applied. **Figure 3** demonstrated the relative importance of 18 variables in cervical cancer risk prediction. Based on this analysis, nine predictors had relative importance greater than one. The use of hormonal contraceptives (years) was identified as the most important risk factor, followed by the number of pregnancies, smoking (years), number of cigarette packets smoked annually, number of sexual partners, the use of an intrauterine device (IUD) (years), number of sexually transmitted diseases (STDs), human immunodeficiency virus (HIV), and age. These nine features were incorporated into the model and cross-validated. In contrast, in the univariate logistic regression, the number of sexual partners was not significantly correlated (p = 0.986) with the occurrence of cervical cancer.

These nine features were incorporated into the model and cross-validated.

### Prediction Performance of the Sampling Method

**Table 2** described the comparative performance scores of different sampling methods using RF. Each sampling model had been verified internally and externally. In the external validation, SMOTE-based RF performed best among all classifiers with an AUC of 0.849 and had the highest score in four of our seven performance metrics. The sensitivity and specificity were 90.9% and 73.1%, respectively, both higher than 70%. The accuracy, precision, F1 score, and F2 score were 74.2%, 18.9%, 0.312, and 0.195, respectively. SMOTE was therefore selected as the imbalance data processing algorithm for the final model.

**Table 2:** Prediction performance of random forest algorithm on different sampling models.

Methods		Cutoff	Accuracy	Precision	Sensitivity	Specificity	F1 Score	F2 Score	AUC
Oversampling	Train set	0.703	0.978	0.749	1.000	0.977	0.857	0.535	0.997
	Test set	0.099	0.660	0.159	**1.000**	0.637	0.275	0.172	0.803
Undersampling	Train set	0.333	0.761	0.191	0.840	0.756	0.312	0.195	0.870
	Test set	**0.343**	**0.743**	0.163	0.727	**0.744**	0.267	0.167	0.739
Both sampling	Train set	0.672	0.947	0.550	0.977	0.945	0.704	0.440	0.988
	Test set	0.191	0.597	0.138	**1.000**	0.569	0.242	0.151	0.784
ROSE	Train set	0.270	0.773	0.171	0.659	0.781	0.272	0.170	0.733
	Test set	0.178	0.632	0.129	0.818	0.619	0.222	0.139	0.745
SMOTE	Train set	0.600	0.952	0.586	0.864	0.958	0.698	0.436	0.968
	Test set	0.268	0.742	**0.189**	0.909	0.731	**0.312**	**0.195**	**0.849**

The portions in bold represent the model is optimal in a single index. ROSE, random oversampling example; SMOTE, synthetic minority oversampling technique; AUC, area under the curve.

Evaluation of the model performance using the receiver-operating characteristic (ROC) (**Supplementary Figure S2**) showed the comparison of the prediction ability of external and internal validation of the model under different sampling models. The curves modeled the sensitivity proportion of actual at-risk women identified at risk of developing cervical cancer to the specificity proportion of identified no-risk women in the models.

### Prediction Performance of 12 Machine Learning Models

**Figure 4:**
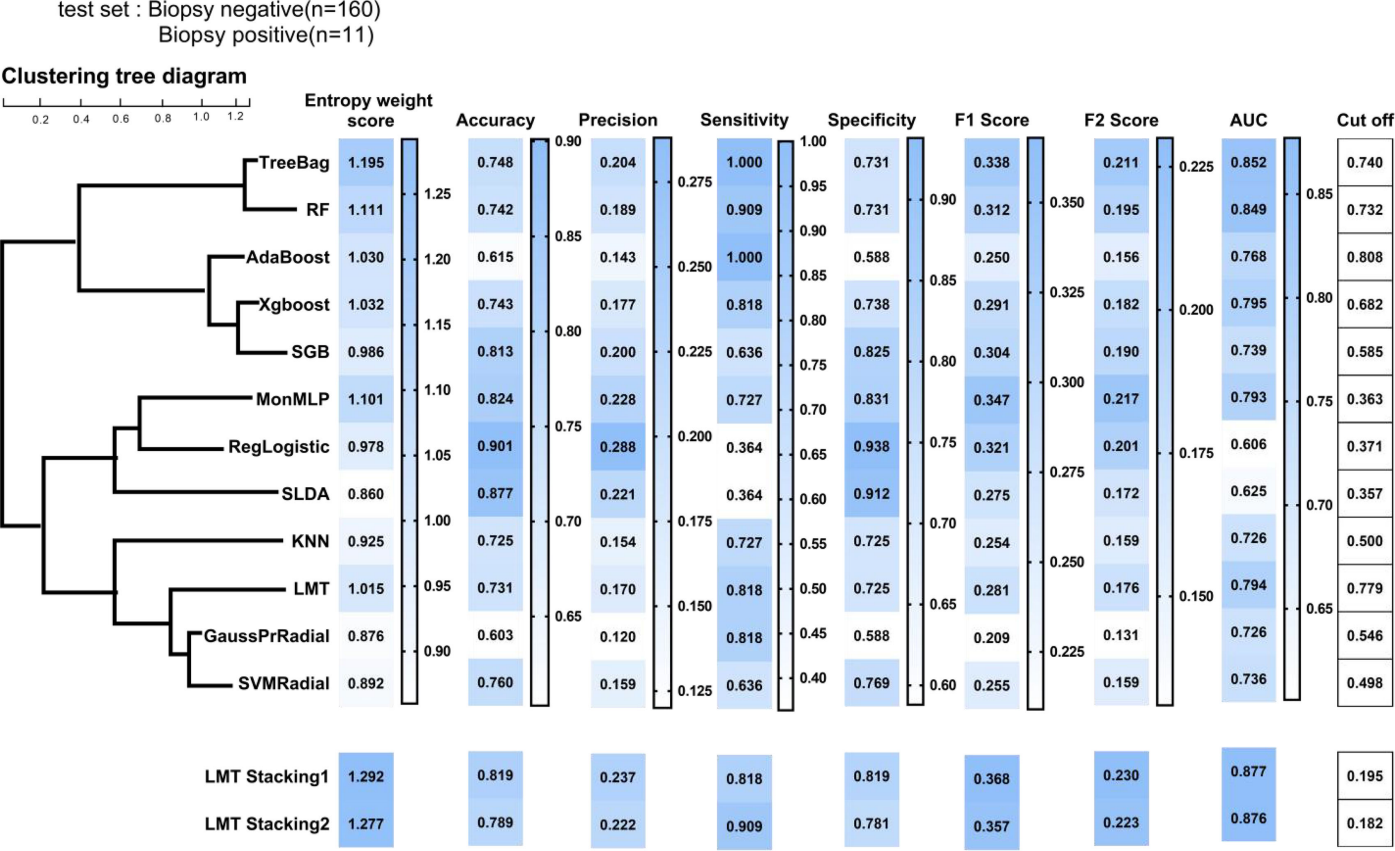
Prediction performance of ML models on the test sample. ML, machine learning; TreeBag, Bagged Classification and Regression Tree; MonMLP, Monotone Multi-Layer Perceptron Neural Network Random Over-Sampling Examples; XGBoost, eXtreme Gradient Boosting; LMT, Logistic Model Trees; RF, random forest; SGB, Stochastic Gradient Boosting; SVMRadial, Support Vector Machines with Radial Basis Function Kernel; KNN, K-Nearest Neighbors; GaussPrRadial, Gaussian Process with Radial Basis Function Kernel; RgeLogistic, Regularized Logistic Regression; SLDA, Stabilized Linear Discriminant; AdaBoost, AdaBoost Classification Trees; AUC, area under the curve.

Toward at-risk patients of cervical cancer classification, **Figure 4** compared the performance metrics of 12 different models. According to the entropy weight score, TreeBag resulted in the best performance, with an AUC score of 0.852 for the test dataset. The sensitivity and specificity were 100% and 73.1%, respectively. Compared to RF, the performance of sensitivity and AUC was improved. As a whole, the tree-based models (TreeBag, RF, Adaboost, XGBoost, SGB, and LMT) performed better than other models, and the performance difference between the models was minor. Additionally, the performance of the deep learning model MonMLP ranked third, with an AUC of 0.793 and sensitivity and specificity of 72.7% and 83.1%, respectively. The MonMLP model was significantly better than other models with top performance in terms of specificity. The tuned parameters of these models were listed in **Supplementary Table S3**.

According to the correlation results of the 12 models (**Supplementary Figure S4**), we divided the 12 models into 4 clusters (**Figure 4**) by using the hierarchical clustering method. The intra-cluster model prediction difference was small, while the inter-cluster model was large. In the first group, TreeBag and RF were included, and the correlation between them was as high as 0.80. Treebag was better than RF in predicting high-risk patients with cervical cancer. According to the hierarchical clustering results, AdaBoost, XGBoost, and SGB belonged to the tree model based on boosting integration and were divided into the second group. The correlation between the three models was greater than 0.50. The best model was XGBoost with an AUC, sensitivity, and specificity of 0.795, 81.8%, and 73.8%, respectively. The third group consisted of the MonMLP model and two simplistic models (RgeLogistic and SLDA). In terms of performance, MonMLP performed better than the other two models. This was partly due to the small number of positive biopsies, and therefore the two simplistic models could not learn enough logical relationships. In the fourth group, only LMT, KNN, GaussPrRadial, and SVMRadial performed well.

### Prediction Performance of Stacking Models

In order to meet the two requirements of the stacking structure for the base classifier and improve the performance (27), we selected an optimal model from each group, namely, TreeBag, XGBoost, MonMLP, and LMT. The performance ranking of those models might be TreeBag > MonMLP > XGBoost > LMT. LMT model was a simpler model based on the Logistic and tree model, with high generalization and strong generalization robustness (26). Therefore, we chose LMT as the second layer structure of stacking (result classifier) and TreeBag, XGBoost, and MonMLP as the first layer (base classifier). Finally, two LMT-stacking models with different tuning parameters were built by training (**Supplementary Table S3**). The AUC, sensitivity, and specificity of the LMT-Stacking1 model were 0.877, 81.8%, and 81.9% (**Figure 4**), respectively, and 0.877, 81.8%, and 90.9%, respectively, for the LMT-Stacking2 model. The difference in AUC between the two models was only 0.1%, and the performance difference was not significant. Similar results were seen in the ROC curves for each of the models, as shown in **Supplementary Figure S5**.

## Discussion

AI and ML algorithms are increasingly used in healthcare to analyze large datasets and perform predictions. However, the use of these algorithms in identifying women at high risk of developing cervical cancer is limited and often based on former generation models, which have more limited accuracy than more advanced algorithms. In this study, we have proposed the use of SIML that integrates multiple algorithms to improve the prediction accuracy.

The findings of this study indicated that various ML algorithms could be used to predict women at high risk of developing cervical cancer based on demographic, behavioral, and clinical data. However, the SIML with TreeBag, XGBoost, and MonMLP as base classifier and LMT as result classifier provided the best overall performance. Compared with the LMT-Stacking1 model, the sensitivity of the LMT-Stacking2 model was highly improved, while the specificity decreased. However, because the data had few positive samples and the sensitivity varied significantly, the performance of the LMT-Stacking1 resulted in a better overall performance because it was more balanced.

### Predictors for Developing Cervical Cancer

According to the feature selection based on RF, hormonal contraceptives (years), the number of pregnancies, smoking (years), the number of sexual partners, the use of IUD (years), and smoking (packs/year) were identified to be the most important influencing factors for the at-risk patient, especially the long-term use of hormone contraceptives. Human papillomavirus (HPV) infection was the leading cause of cervical cancer (31). According to Cox (32), the risk of developing an HPV infection was not only related to age but also increased with the increasing number of sexual partners, highlighting the need to improve awareness and improve vaccination campaigns. Co-infection with HIV might impair the ability of the immune system to control HPV infection. Additional risk factors included smoking, high parity, and long-term use of hormonal contraceptives (31). Exogenous hormones had been considered as auxiliary factors in the pathogenesis of cervical cancer caused by HPV. If the HPV-positive women took the hormone contraceptives for a long time, the risk of cervical squamous cell carcinoma tripled (33). Smoking was related to the development of squamous cell carcinoma and was an auxiliary factor and primary carcinogen in the development of cervical cancer (34). The use of IUD could create a potential malignant focus close to the cervical canal, eventually creating a transformation zone whereby preneoplastic lesions arise. The transformation zone was both targeted by HPV and a major effecter and inductive site for cell-mediated immune response (35).

### Machine Learning and Cervical Cancer

Most studies on cervical cancer made use of ML to predict survival in cervical cancer (36). Although some studies had used generalized estimating equation regression models to predict the early risk probability of developing cervical cancer (34), their prediction accuracy remained limited. Our ML model utilized more features and could, therefore, improve the prediction accuracy. The Pittsburgh cervical cancer screening model consisted of 19 variables, including cytological examination and HPV test results. The incidence of cervical cancer was predicted by combining the case results, detailed medical history [including gender, HPV vaccination status, menstruation, contraception history, age, and race (37)]. The model could be used for risk stratification of patients only after screening. The advantage of our proposed model was that it provided a simple tool to identify high-risk groups before screening by combining behavioral data provided by patients with clinical data.

## Limitations

The main limitation of this study was the limited sample size and population coverage. Compared with deep learning, SIML had the advantage of being suitable for small sample data, which only needed 80–560 samples. The specific sample size required depended on the dataset and sampling method (38). Therefore, the sample size in our study was sufficient to build a model. If the overall sample size was increased, the performance of the model could be improved significantly. Additionally, some potentially important parameters, such as previous screening information, were not considered in our study. Data on variation in behavioral patterns over time were not available, and therefore, we could not establish their impact on the model. Moreover, samples were obtained from the same institution, limiting the generalizability of the model. Although we used a combination of internal and external validation, we recommend the use of external datasets to further test the performance of this model.

## Conclusions

This study shows that SIML can be used to accurately identify women at high risk of developing cervical cancer and performed better than other ML algorithms. This model could be used to personalize the screening program by optimizing the screening frequency and improving the care plan in high- and low-risk women based on their demographics, behavioral patterns, and clinical data. This will eventually reduce unnecessary screening in low-risk groups and hence reduce the screening costs.

## Data Availability Statement

The original contributions presented in the study are included in the article/**Supplementary Material**. Further inquiries can be directed to the corresponding authors.

## Author Contributions

BP and LS contributed to the protocol design. LS and QZ analyzed data. XL, YG, and QC contributed to the interpretation results. LS drafted the article, and LY proofread the article. BP, LT, and ZL revised the final version and are guarantors of this article. All authors made substantial contributions to the paper and read and approved the final article.

## Funding

This study was funded by the National Key Research and Development Program of China (No. 2018YFC1311706).

## Conflict of Interest

The authors declare that the research was conducted in the absence of any commercial or financial relationships that could be construed as a potential conflict of interest.

## Publisher’s Note

All claims expressed in this article are solely those of the authors and do not necessarily represent those of their affiliated organizations, or those of the publisher, the editors and the reviewers. Any product that may be evaluated in this article, or claim that may be made by its manufacturer, is not guaranteed or endorsed by the publisher.

## Supplementary Material

Supplementary Figure 1 Visualization results before and after missing values was filled. The part of red color is the missing value, with each column as the standard. The larger the value is, the darker the color is. On the contrary, the smaller the value is, the lighter the color is.

Supplementary Figure 2 Receiver operating characteristic curves for Random Forest prediction performance of difference Sampling models.

Supplementary Figure 3 Receiver operating characteristic curves for 12 ML models.

Supplementary Figure 4 Correlation coefficient diagrams of 12 ML models.

Supplementary Figure 5 Receiver operating characteristic curves for LMT-stackingmodels.
