# Supplementary figures and images for: Optimization of Cervical Cancer Screening: A Stacking-Integrated Machine Learning Algorithm Based on Demographic, Behavioral, and Clinical Factors
Source: Front Oncol. 2022 Feb 15;12:821453. doi: 10.3389/fonc.2022.821453 (PMC8886038; doi:10.3389/fonc.2022.821453)

**A. Before the missing value was filled**

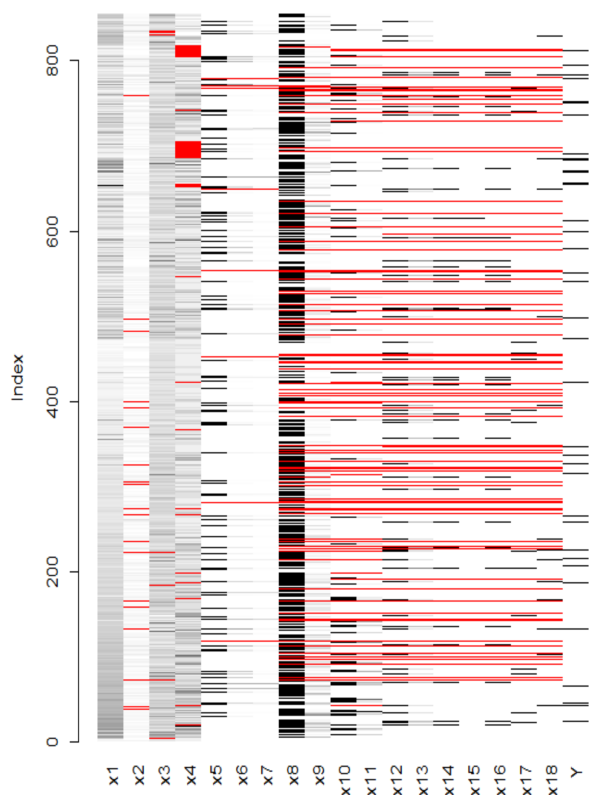

**B. After the missing value was filled**

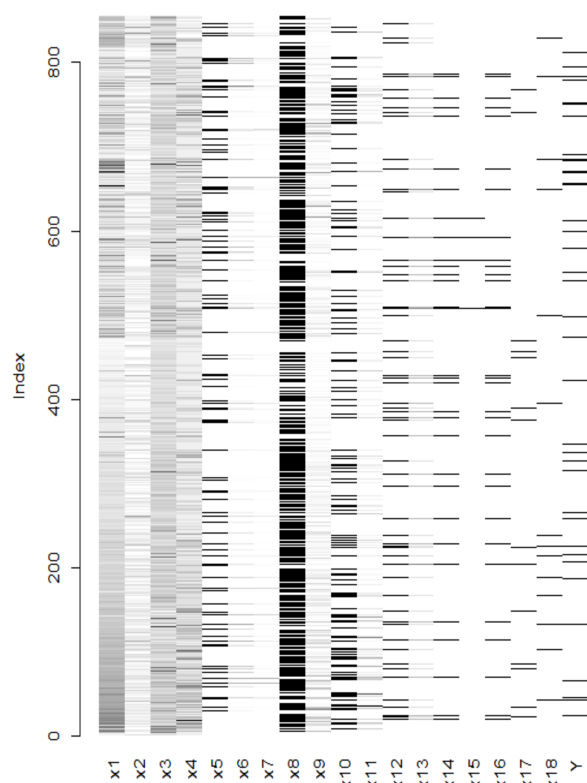

Supplement: Supplementary Figure 1 — Visualization results before and after missing values was filled. The part of red color is the missing value, with each column as the standard. The larger the value is, the darker the color is. On the contrary, the smaller the value is, the lighter the color is. [file Image_1.pdf]

**A.training sample**

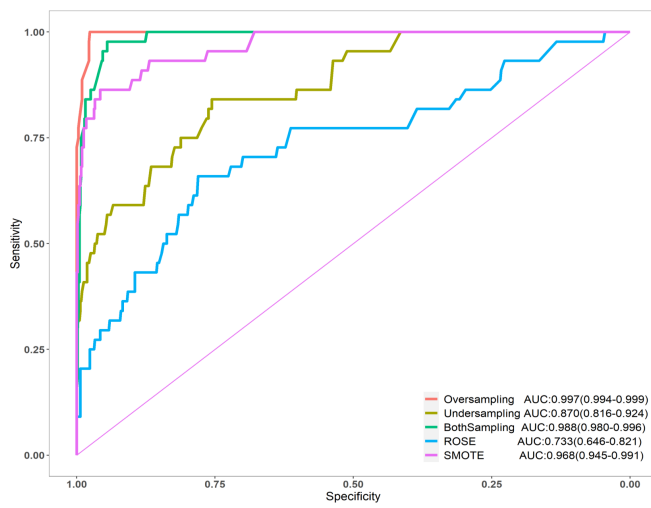

**B.test sample**

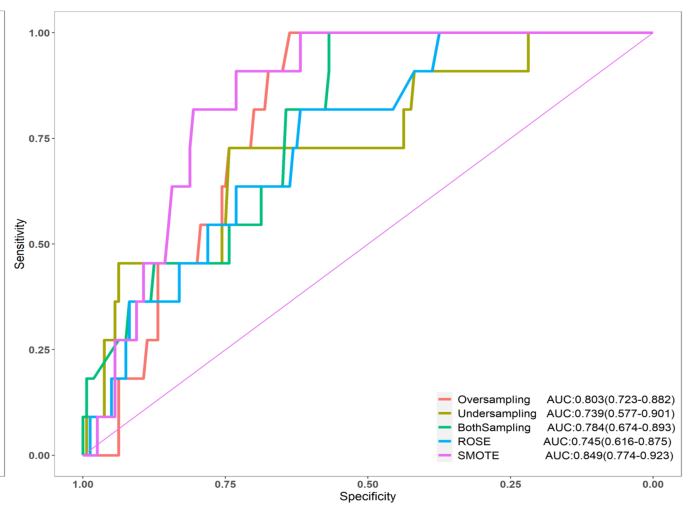

Supplement: Supplementary Figure 2 — Receiver operating characteristic curves for Random Forest prediction performance of difference Sampling models. [file Image_2.pdf]

**A.training sample**

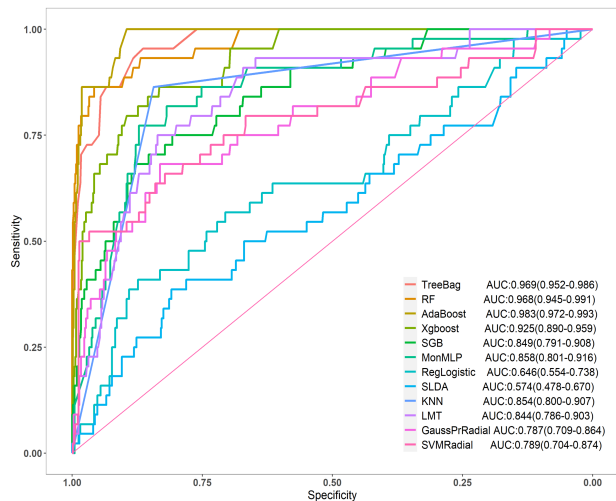

**B.test sample**

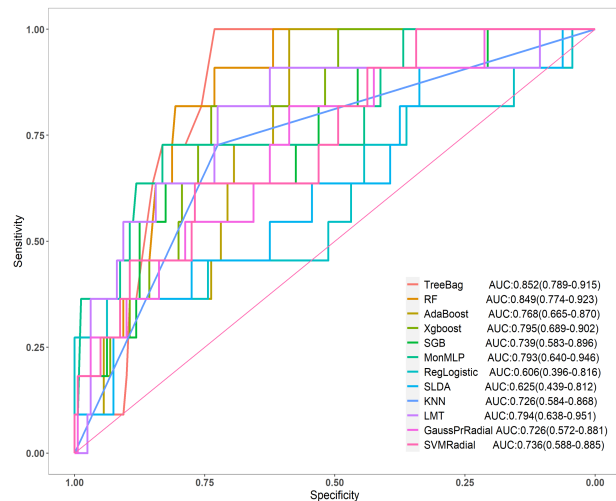

Supplement: Supplementary Figure 3 — Receiver operating characteristic curves for 12 ML models. [file Image_3.pdf]

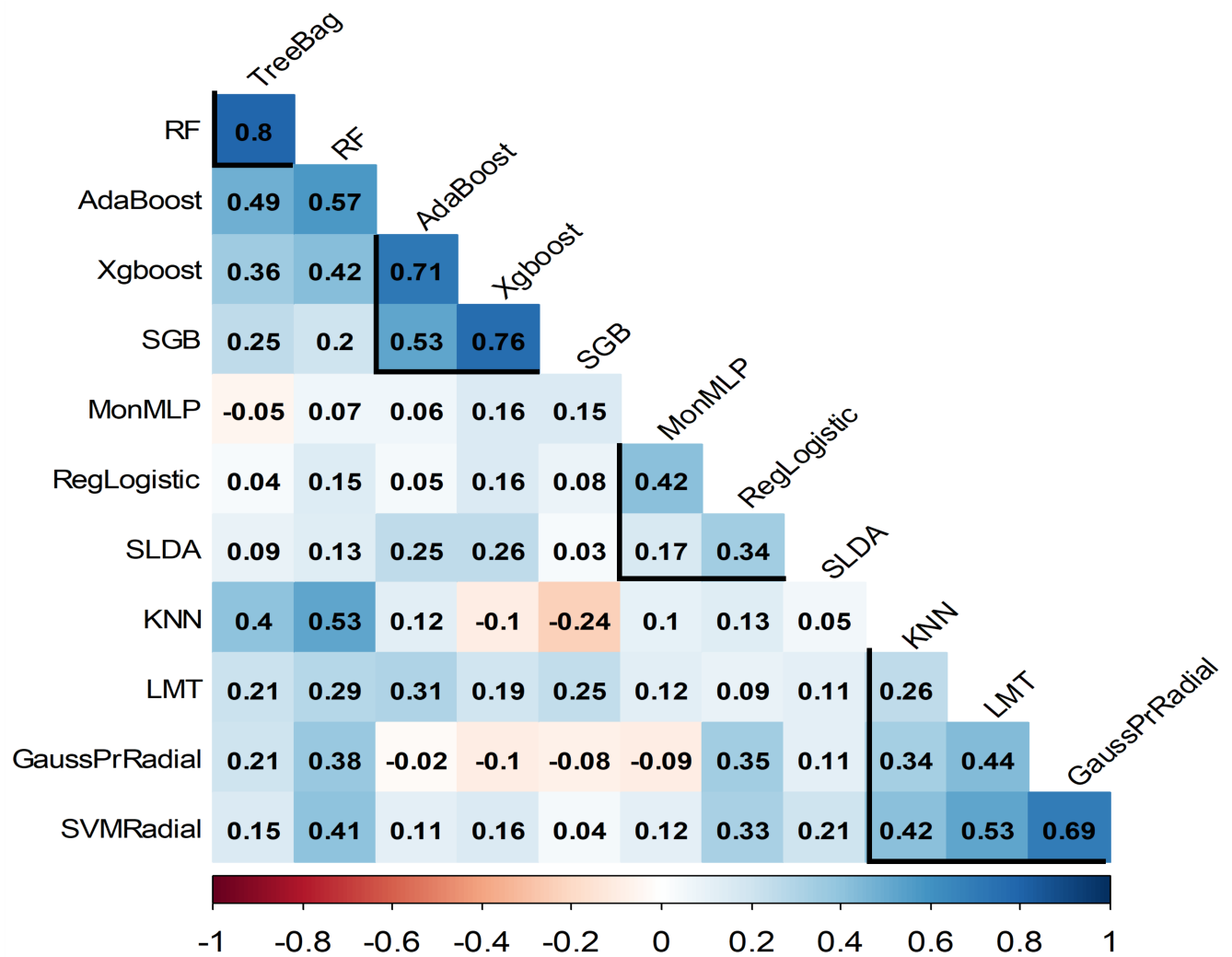

Supplement: Supplementary Figure 4 — Correlation coefficient diagrams of 12 ML models. [file Image_4.pdf]

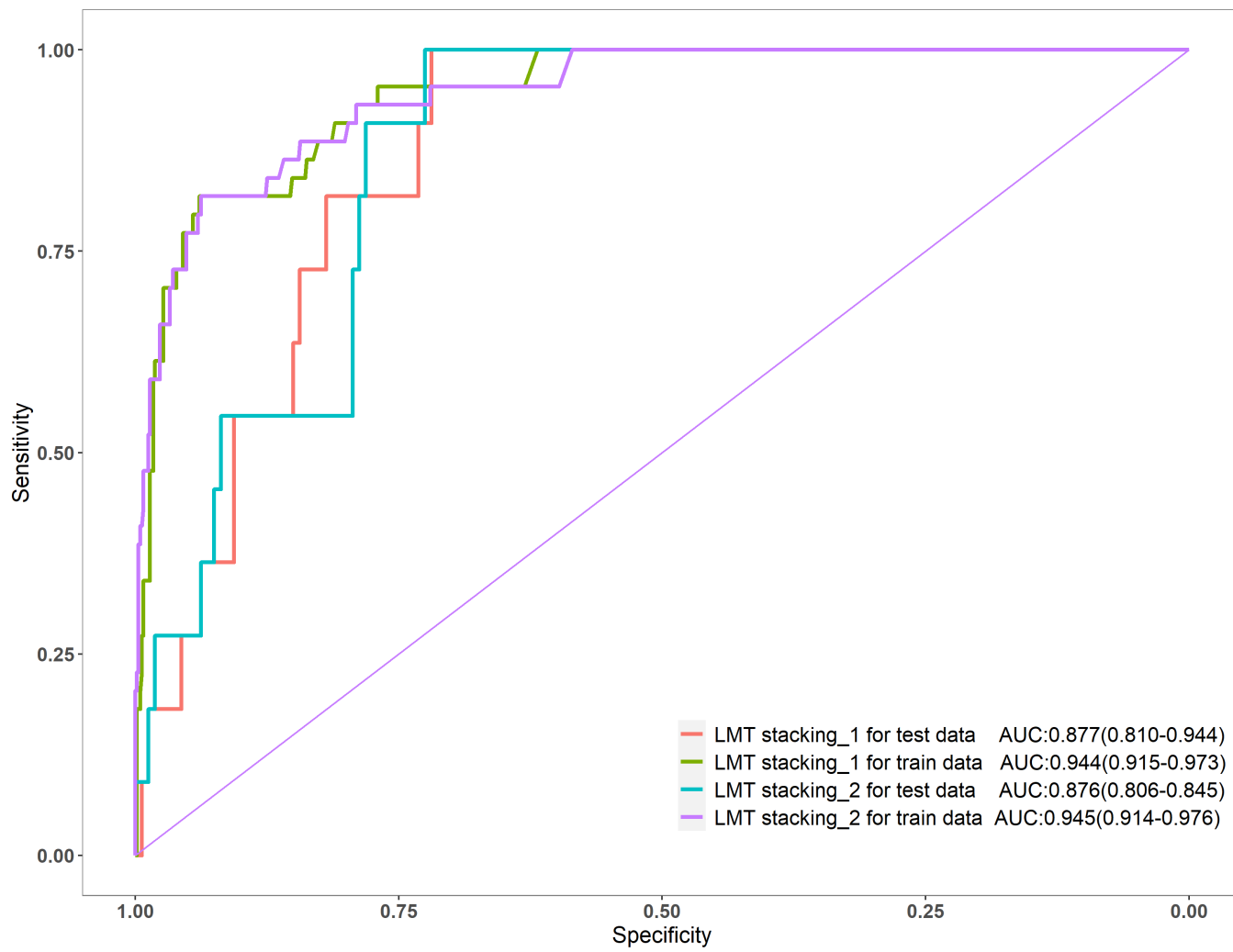

Supplement: Supplementary Figure 5 — Receiver operating characteristic curves for LMT-stackingmodels. [file Image_5.pdf]
